# Virtual Reality in Cancer Care: Enhancing Knowledge and Reducing Anxiety about Chemotherapy among Patients and Caregivers

**DOI:** 10.3390/ijerph21091163

**Published:** 2024-09-01

**Authors:** Melissa K. Thomas, Abolfazl (Abel) Jarrahi, Lauren Dennie, Sam Scott, Ted Lau, Annika Johnson

**Affiliations:** 1 Heritage College of Osteopathic Medicine, Ohio University, Athens, OH 45071, USA; ld464622@ohio.edu (L.D.); tl489319@ohio.edu (T.L.); annikajohnson240@gmail.com (A.J.); 2 VR Medical Solutions, Athens, OH 45701, USA; jarrahi@ohio.edu; 3 College of Arts and Sciences, Ohio University, Athens, OH 45701, USA; ss288720@ohio.edu

**Keywords:** virtual reality (VR), chemotherapy, cancer care, patient education, anxiety reduction, immersive technology, VR simulation

## Abstract

Virtual reality (VR) technology has evolved from entertainment to significant applications in healthcare and education. Despite its potential, there is limited research on the role of VR in cancer care. This study investigates VR’s ability to simulate the chemotherapy process, aiming to enhance patients’ knowledge and mitigate anxiety associated with chemotherapy. Utilizing a two-arm, mixed-methods pre/post-survey design, the study measured changes in patients’ anxiety and knowledge before and after exposure to a VR simulation. Participants (*n* = 267) engaged with VR simulations or interactive 360-degree videos depicting the chemotherapy process. Data analyses revealed a significant median increase in chemotherapy knowledge post-exposure to the VR content (z = 12.511, *p* < 0.001). Demographic factSors significantly influenced perceptions of VR realism and usefulness (*p* < 0.05). Additionally, VR exposure was correlated with reduced anxiety levels and improved treatment expectations (*p* < 0.05). Participants with higher post-understanding chemotherapy scores considered VR a useful tool for managing anxiety about chemotherapy and recommended VR for other medical procedures (*p* < 0.001). These findings underscore VR technology’s potential as a valuable tool in cancer treatment, suggesting it can enhance patient education and reduce anxiety, thereby improving patient outcomes during cancer therapy.

## 1. Introduction

Virtual reality (VR) is an advanced technology that offers immersive virtual environments, allowing users to delve into 360-degree simulations and providing a spherical visual experience unattainable through traditional media [1,2]. This technology’s hallmark lies in its ability to convincingly transport users into simulated settings using headsets to provide a sense of immersion [3,4]. VR differs from Augmented Reality (AR), which overlays digital elements onto the real world without fully replacing it. While definitions of VR and AR can vary across fields in medical education and patient care, VR is distinct because of the complete immersion it provides [2,5]. A key component of VR is the use of the VR headset in order to engage fully with the digital space. AR often has a digital interface that allows users to interact with the AR content, often through gestures, voice commands, or touchscreens [2,5].

### 1.1. Virtual Reality in Healthcare

VR has expanded beyond home entertainment and is now used in educational settings as well as a tool to bridge gaps in professional fields [3,4]. The concept of “pre-reality” in VR enables users to virtually experience real-life settings before encountering them in person [6]. Pre-reality is often designed to function as a tool to prepare for anticipated high-stress or emergency situations where users would benefit from forethought and reflection [7]. This immersive approach is particularly effective in helping individuals become familiar with their surroundings, amenities, and necessary pathways within a given environment. Unlike traditional preparation of sharing a paper map or being given verbal descriptions, VR offers a low-stakes pathway for individuals to navigate a new scenario. This familiarity can significantly reduce anxiety and improve preparedness for real-life experiences [8].

In healthcare, the presence of VR continues to grow, both in medical education and patient care [9,10,11]. Recent studies have showcased VR’s transformative impact on medical education by enhancing skills, knowledge, and clinical decision-making through interactive training modules [3,4,12]. These modules empower clinicians to use the skills learned through VR in their practice [4]. Medical students learning in a VR immersive environment, compared to those using screen-based learning, demonstrate higher knowledge retention [4]. Moreover, the use of VR in a learning environment has been shown to increase the empathy of healthcare providers and participants experiencing certain health conditions for the first time “to see what it is like in someone else’s shoes” (p. 80) [13].

### 1.2. VR’s Impact on Emotional Support and Anxiety Reduction

VR is used in patient care to foster patient empowerment and control [14,15,16,17]. An example in cancer care utilized a simulated game on an iPad to teach patients about chemotherapy and self-management of their symptoms at home [17]. After playing the simulated chemotherapy patient game, participants reported increased confidence in managing their symptoms. This simulation allowed patients to feel more in control over their symptoms. Throughout the cancer continuum, studies highlight VR’s efficacy in distracting chemotherapy patients from treatment-related symptoms, such as anxiety and nausea [18,19,20]. Using VR during chemotherapy allows patients to become immersed in their simulated surroundings, diverting them from their pain, nausea, and anxiety during their treatment [6,21,22,23].

VR is also used to induce relaxation through simulated scenarios like nature walks or scenes of streams, lakes, or forests [24,25,26,27]. As urbanization limits exposure to nature, such immersive experiences have become increasingly important for patients [12,28,29]. VR’s effectiveness lies in its ability to serve as a distraction technique to alleviate pain and anxiety during biopsy procedures [30]. By engaging patients with visual and auditory stimuli, VR helps divert attention from discomfort to the immersive experience. VR can also be used as a motivational tool, providing task-oriented programs that enhance patients’ quality of life and functionality [20]. These programs allow patients to focus on enjoyable stimuli rather than distressing symptoms associated with cancer treatment. Overall, VR fosters psychological safety through pleasant nature simulations, encouraging participant engagement and self-directed learning [4].

### 1.3. The Unique Advantages of VR for Patient Education

Utilizing recorded technology designed for VR experiences creates numerous opportunities to reach a broader and more diverse audience with customized content, such as translations into different languages and personalization for specific hospitals and facilities. This type of recorded explanation enables patients and caregivers to revisit the treatment information provided to them. It also fosters a common understanding or consensus of chemotherapy among patients, caregivers, and medical staff. The use of recorded technology designed for VR allows the recorded content to address patients’ knowledge gaps in terms they understand. According to the communication accommodation theory, people adjust their speech based on their audience [31]. One study found positive indirect effects on participants’ health behavioral intentions and their evaluations of physicians who used simpler, more understandable language [32]. The use of analogies or metaphors, for instance, significantly improved patient comprehension. This evidence supports the concept of a universal language for communicating chemotherapy information through VR to enhance understanding.

VR training in medical education offers cost-effective patient care solutions [24,33,34]. VR’s immersive environments can eliminate the necessity for costly training equipment and the presence of an instructor [33]. In addition to reducing training equipment costs, VR enhances teaching efficiency by delivering clear and concise information that can be replayed and reviewed as required while maintaining consistent communication of information. A recent pilot study showcased the promising effects of the use of head-mounted displays and also video streaming in increasing empathy among healthcare providers, which also adds to the cost-effective benefit of VR training in places where budgets are limited or access to technology is less accessible in broadband deserts found mostly in the rural and Appalachian regions of our study area [35,36].

A cancer diagnosis imposes significant emotional strain on both patients and caregivers, creating a need for technological solutions to help reduce anxiety and improve coping strategies [28,37]. Additionally, patients often receive insufficient information about what to expect from chemotherapy treatments, leading to increased fear and uncertainty [38,39]. While VR is predominantly used as a distraction tool in medicine to alleviate pain and anxiety, its potential as an educational tool in cancer treatment remains underexplored [6,9,10,11,28]. For example, in breast cancer patients undergoing radiation therapy, a VR educational intervention was developed to increase knowledge of radiation therapy treatment and decrease patient anxiety [9]. Patients who viewed the VR simulation reported a deeper understanding of radiation therapy and decreased anxiety in the short term [9]. Similarly, in breast cancer patients undergoing chemotherapy, VR was utilized as a psycho-social intervention protocol to decrease anxiety and depression [40].

This pilot study explores the immersive potential of VR in allowing caregivers to attain the same level of understanding about chemotherapy as patients before treatment, facilitating better preparation and support during and after the treatment process. By visualizing and explaining chemotherapy procedures through VR, the study aims to clarify the treatment process and empower patients and caregivers, ultimately improving the oncology healthcare experience. We hypothesize that after viewing a VR simulation of a typical chemotherapy appointment, there will be an increase in knowledge and a decrease in anxiety regarding the chemotherapy process.

## 2. Materials and Methods

### 2.1. Context

This study was inspired by the firsthand experiences of one of the co-investigators (AJ) who, during his cancer treatment in 2020, identified significant gaps in patient education regarding chemotherapy. Traditional methods, often reliant on dense pamphlets filled with medical jargon, proved insufficient and overwhelming. Driven by his experiences, the co-investigator founded a startup dedicated to leveraging VR technology to address these educational shortcomings in healthcare [12,14,41]. The company collaborates closely with medical practitioners and institutions to develop custom VR experiences to cater to the unique challenges patients and their caregivers face. The project, “Chemotherapy Unveiled”, aims to enhance patient understanding and reduce anxiety through immersive virtual tours of medical processes [42].

Traditional methods of cancer information dissemination, like pamphlets, often miss the mark in patient education due to their complex language, while the overwhelming and conflicting information online tends to heighten patient anxiety [43]. This, in turn, contributes to the widespread issue of depression and anxiety among cancer patients [44], emphasizing the need for better educational and emotional support systems. Our project addresses this gap by using virtual reality experiences to improve patient understanding and alleviate anxiety during chemotherapy, offering a more effective approach to patient care.

### 2.2. Video Production

The VR experience was recorded using an Insta360 One camera attached to a baseball cap to show the perspective from a patient’s point of view. The person operating the camera acted as a patient while wearing a Zoom H3 audio recorder on their chest to capture sound, enhancing the realism of the experience. The video guided viewers through each step of the chemotherapy process, starting from checking in at the hospital, interacting with nurses, and touring the chemotherapy facility. Nurses provided instructions on where treatment takes place, answered questions posed by the patient, and highlighted some of the amenities available at the hospital to create a soothing and comfortable environment for patients receiving treatment. Hospital staff were advised to interact with the camera operator as they would with a patient. The final edited video lasted 18 min and focused on showcasing patient interactions and informative content.

### 2.3. VR Platform and Distribution

The edited video was exported in a 360-degree format with ambisonic audio suitable for VR headsets. For remote viewing, the video was hosted on a platform capable of streaming 360-degree content, allowing participants to navigate the video using their own VR headsets or through standard devices such as smartphones, tablets, or computers. To explore the simulated environment, participants using a desktop could use their mouse to view their surroundings. Participants using a handheld device could rotate their device to explore the environment. For in-person participants, the video was loaded onto 25 Pico VR headsets equipped with LookandPlay software (v.5.1) to facilitate seamless viewing without the need for manual navigation.

### 2.4. Measures

The pre-intervention survey consisted of 20 multiple-choice questions with either yes/no or four- to five-response options. Administered via the Qualtrics platform, the pre-intervention collected demographic data (gender, ethnicity, employment status, education level), mode of participation (in-person or virtual), technology used for virtual simulation, confidence in understanding new technology, knowledge of the chemotherapy process, and any prior chemotherapy experience for the participant or their loved ones. These questions established a baseline understanding of participants’ perceptions of the chemotherapy process before viewing the simulation. The survey questions were adapted from standardized tools, including the State-Trait Anxiety Inventory (STAI) and Likert scales, to assess both anxiety levels and knowledge improvement. The questions were carefully worded and modified to ensure relevance to the study’s focus on chemotherapy education and anxiety reduction [45].

The post-intervention survey contained 15 questions and evaluated changes in participants’ understanding of the chemotherapy process following the intervention. The survey included both multiple-choice and open-ended questions to capture participants’ knowledge, expectations, and anxiety levels regarding chemotherapy after the intervention. Participants were asked if the simulation influenced their understanding and whether they believed it could be a useful tool for individuals about to undergo chemotherapy. The post-intervention also assessed the realism and quality of the simulation video and solicited feedback on areas for improvement.

### 2.5. Participants

Participants were recruited through community advertisements, social media platforms, and healthcare facilities. Interested participants were guided to an online screening questionnaire for eligibility assessment. The study recruited a sample of adult participants who met the following criteria: (1) aged 18 years or older and (2) able to understand English. We enrolled only English-speaking participants in this study due to the specific demographic and geographic characteristics of our region. As a pilot study conducted primarily in the Midwest of the United States, the majority of participants were native English speakers. Limiting enrollment to English speakers allowed us to streamline the study process and focus on the feasibility of the intervention within the local population with English-speaking actors and language in the VR experience. Eligible participants received information about the study’s objectives, procedures, potential advantages and disadvantages, confidentiality protocols, and their right to opt out at any stage. The study was approved by the university’s Institutional Review Board as an exempt study, requiring no signature given anonymous participation in the study. Participants were required to click on a survey question to confirm they had read and acknowledged informed consent before they could proceed with the rest of the study.

### 2.6. Procedure

Participants who granted consent completed a survey before the intervention. They had the option to participate virtually or in person in a university setting, depending on their preference and accessibility. Those who chose to participate in person used the VR headset to engage in the VR experience. Participants who contributed virtually had the option to use their own VR equipment or engage through a 360-degree video on a computer or handheld device. Participants in the VR group watched a 30-min VR simulation session in a controlled laboratory environment that included a quiet, large meeting room with no outside distractions. The VR simulation showed a first-person perspective of a patient learning about chemotherapy. Participants in the virtual video group received a link to the same immersive video, where they were able to interact with it using the mouse or touch control. When participants did not elect to use the VR headsets, they were engaged in AR as they could still interact in their environment (360 view through a touchscreen) and get the realism of the first day of chemotherapy. These participants could watch the video at their convenience. Following the VR session or video viewing intervention, participants were asked to complete a post-intervention survey online. After the study was completed, in-person participants received a debriefing session about the study’s purpose. All participants were offered the opportunity to ask questions or provide feedback via in-person or email correspondence.

Two important ethical considerations were taken into account when designing the study. Participants might feel uneasy or uncomfortable, especially when sharing experiences related to medical treatments or hospitals. To address this discomfort, participants were assured that their responses would be kept anonymous and confidential. They were informed they had the right to withdraw from the study at any point. Additionally, there was a chance that participants could experience motion sickness during the VR simulation. Participants had the freedom to end the session if they felt unwell and were given the option to watch a recorded video instead. Throughout the VR experience, participants received clear guidance and assistance.

### 2.7. Data Analysis

We utilized a mixed methods approach to examine the information collected from both the pre-intervention and post-intervention surveys. We used Chi-Square and Spearman Rho tests to investigate the connections between participant demographics, cancer, technology usage, and VR variables. The sign test was used to assess changes in participants’ knowledge of chemotherapy and their anxiety levels post-intervention.

We also conducted a thematic analysis to pinpoint and study recurring themes and patterns found in the open-ended responses. We utilized an inductive approach to identifying themes and employed the six-phased model introduced by Braun and Clarke [46]. Three of the co-authors independently applied concept-driven coding methods to analyze these responses before discussing the outcomes together until reaching an agreement on all coded data utilizing Microsoft Excel (version 2407) to capture the individual coder’s responses and final responses to each participant’s open-ended responses. (MT, AJ, SS). The qualitative analysis was guided by the 21 items of the Standards for Reporting Qualitative Research (SRQR) [47]. By delving into participants’ personal experiences, we aimed to gather insights into how the intervention influenced their comprehension of chemotherapy and their emotional well-being.

## 3. Results

The majority of participants in the study were aged 18 to 34 years (66.3%), while only nine individuals were 65 or older (3.4%) (Table 1). Regarding cancer history, 8.6% of participants were identified as survivors, while 91.4% had no personal cancer history. However, a significant portion (82.0%) reported knowing someone who had experienced cancer. Approximately half (48.7%) of the participants held graduate degrees, and around 17% reported either a high school or post-graduate education. A larger segment of participants were employed (78.7%). The majority of the participants identified as White (76.7%), followed by African American (13%), Hispanic (4.9%), and Other (13%). A total of 189 (71%) were female, 25.6% were male, and 1.1% identified as transgender. The most common technology platform used to view the intervention was via cell phones (43.8%), followed by laptops (33.7%) and VR headsets (10.5%). Only 8.2% used desktop computers, and 3.7% used tablets for viewing.

An exact sign test was conducted to determine the effect of the VR experience on understanding the chemotherapy process. A total of 267 participants interacted with the video either at home or in person using VR headsets. A five-point Likert scale was administered before and after viewing the video, and it included the following responses to the question “How well is your understanding of the chemotherapy process?”: 1, Very Poor; 2, Below Average; 3, Average; 4, Above Average; 5, Excellent. Viewing the VR video led to an increase in the post-intervention understanding scale in 194 participants, whereas only 13 listed a lower understanding score after the viewing. There was a statistically significant median increase in understanding from 1 before viewing the VR video to 4 after viewing compared to the pre-intervention scores (3), *z* = 12.511, *p* < 0.001.

An exact sign test was also conducted to determine the effect of a VR experience on one’s belief in VR as a useful tool in managing anxiety about chemotherapy. A five-point Likert scale was administered before and after viewing the video and included the following responses to the question “To what extent do you feel that VR could be a useful tool in managing anxiety about chemotherapy?”; 1, Not at all; 2, Somewhat; 3, Moderately; 4, Very Much So. Of the 267 participants recruited for the study, the viewing of the VR video led to an increase in the post-intervention anxiety scale in 79 participants, whereas 65 listed a lower anxiety score after the viewing. There was no statistically significant median increase in anxiety reduction (0) after viewing the VR video (3) compared to the pre-intervention scores (3), *z =* 1.083, *p* = 0.279. It should be noted that participants already had a very positive belief in VR serving as a useful tool in managing anxiety prior to watching the VR simulation, with over 75% (201) stating “moderately” or “very much so”. Additionally, we noted a slight change in language in the post-intervention anxiety question that asked, “How much did the VR experience help to reduce anxiety about chemotherapy?” with different responses than the pre-intervention question: 1, Not at all; 2, Very little; 3, Somewhat; 4, To a great extent. The already high belief in VR serving as a tool for managing anxiety may not have provided enough initial variability to significantly change the anxiety scores following the VR viewing.

We investigated VR encounters correlated with demographic factors, as outlined in Table 2. Generally, men tended to view VR more favorably than women (*p* = 0.022), with 81.8% of men perceiving VR positively compared to 66.8% of women. A clear link was noticed between responses to VR and ethnicity among Black participants (*p* = 0.022). Only half of the Black individuals reported changes in their emotional experiences, in contrast to 69.7% of non-Black individuals. White participants considered VR more realistic (*p* = 0.030) and more useful compared to non-White participants (*p* = 0.049). It is important to note that all individuals in the virtual reality video were White, filmed at a rural hospital center situated in a county where 93 percent of the population is White. We also found a positive correlation between the perceived realism of VR experiences and age, indicating that older individuals viewed VR as more realistic (*p* = 0.032). These findings shed light on the connection between VR experiences and demographic variables such as gender, ethnicity, and age, offering insights for future research and advancements in the field of VR as a tool in medical settings.

We also investigated the association between virtual reality (VR) experiences and various post-intervention variables (Table 3). We found a direct correlation between participants’ perception of the VR experience and their understanding of the content post-intervention. Those with higher post-understanding rated their VR experience more positively (*p* = 0.048). Similarly, a positive association existed between perception and post-anxiety, suggesting that those who rated their perception above average were more likely to report reduced anxiety about chemotherapy treatment (*p* < 0.001). Furthermore, we found a direct relationship between perception and post-expectation (“How much do you feel that the VR tour helped to manage your expectations about the chemotherapy process?”), indicating that participants with a greater understanding post-intervention tended to have more positive perceptions of the experience (*p* < 0.001). Regarding emotional responses, a direct correlation was found between emotional changes and post-understanding, post-anxiety, and post-expectation. Participants with higher post-understanding were more likely to report changes in their emotional and physical experiences post-intervention (*p* = 0.009). Additionally, those who reported a reduction in anxiety post-intervention were more likely to indicate positive emotional changes (*p* = 0.002).

The perceived usefulness of VR showed a significant correlation with post-understanding, post-anxiety, and post-expectation (Table 4). Participants with a better understanding post-intervention were more likely to consider VR as a useful tool in managing anxiety about chemotherapy (65.4% vs. 29.1%, *p* < 0.001). Additionally, participants who felt that the VR experience provided a clearer image of chemotherapy were more likely to rate higher in post-understanding (30.8% vs. 1.9%, *p* < 0.001) and reported reduced anxiety post-intervention (100% vs. 96.4%, *p* < 0.001). Feelings of control during the VR experience were positively associated with higher post-understanding, post-anxiety, and post-expectation scores. Participants who felt more in control were more likely to have a better understanding post-intervention, reported reduced anxiety, and had higher expectations regarding VR effectiveness (*p* < 0.001). Additionally, those who rated higher in post-understanding were more likely to recommend VR for other medical procedures (96.2% vs. 58.5%, *p* < 0.001). VR realism perception also showed correlations with post-understanding, post-anxiety, and post-expectation (*p* < 0.001), suggesting that participants with higher realism perception were more likely to have positive post-intervention responses.

We included four open-ended questions in the post-intervention survey that asked the following: (1) Did the video change your understanding of the physical and emotional experience of chemotherapy? If yes, please explain how; (2) How did the VR experience make you feel more equipped to handle anxiety-provoking situations, such as the chemotherapy process?; (3) Were there any parts of the VR tour that you found particularly helpful or enlightening?; and (4) Were there any parts of the VR tour that you found confusing or inaccurate? The qualitative data analysis for the study focused on understanding the experiences and perceptions of participants using two VR modalities: 360-degree video and VR headset. Three co-authors categorized responses into several key themes:

- VR Experience: This theme captured the overall user experience, including concepts like the first-person perspective, the friendliness and support of the staff, the realism of the VR content, side effects such as dizziness, and specific mentions of the nurse.
- Knowledge: information conveyed through the VR experience, including the process of chemotherapy, explanation of treatment steps, symptoms and side effects, question and answer sessions, and the elimination of myths and misconceptions about chemotherapy.
- Environment: the physical setting portrayed in the VR experience, such as interactions prior to meeting the nurse, the surroundings and space, and resources provided (e.g., snacks, blankets, seat warmers).
- Emotional: the emotional impact of the VR experience on participants, particularly in terms of relieving or inducing anxiety.
- Improvements: areas for enhancement in the VR experience, including technical aspects of the VR video (e.g., audio quality, video clarity, camera stability) and content-related improvements (e.g., tailored chemo experiences, simulations of actual treatment procedures).
- Overall Reactions: This theme summarized the overall positive and negative reactions of participants to the VR experience.

A listing of themes, definitions, and exemplars can be found in Table 5.

Table 6 compares qualitative codes between the two VR viewing experiences: 360-degree video via YouTube (*n* = 241) and the VR headset (*n* = 26). Key findings include a higher incidence of VR side effects/dizziness reported by VR headset users (23.1% vs. 1.7%, *p* < 0.001) and a mention of increased anxiety (15.4% vs. 2.9%, *p* = 0.015). The mention of knowledge (e.g., the chemo process, explaining chemotherapy/treatment steps, symptoms/side effects, answering questions) was high in both groups (78.4% for 360-degree and 88.5% for VR headset, *p* = 0.229). The mention that the video relieved anxiety was high for both groups, with 85.9% for 360-degree and 96.2% for VR headset users, though not statistically significant (*p* = 0.220). The VR headset group reported more areas for improvement (73.1% vs. 31.5%, *p* < 0.001), especially in VR video quality (53.8% vs. 12.4%, *p* < 0.001). Overall, positive experiences were high for both groups (91.7% for 360-degree and 100% for VR headset, *p* = 0.235), but the mention of negative experiences was significantly higher for VR headset users (76.9% vs. 32.8%, *p* < 0.001). In the analysis of qualitative themes linked to post-understanding, several notable connections were detected (Table 6).

Concerning the “Process” theme, e.g., the process of chemotherapy (checking in, explaining the procedure, sitting down with the nurse, etc.), a greater proportion of participants with above-average (64.1%) or excellent (20.3%) post-understanding levels reported experiencing the process during the VR session (*p* = 0.016) (Table 7). Participants with above-average (60.3%) or excellent (20.5%) understanding and those who reported more positive perceptions of the chemotherapy process (72.5% vs. 27.5% stays the same) were more inclined to report experiencing emotions during the VR session compared to those with average understanding (*p* = 0.033 and *p* = 0.043, respectively). Concerning anxiety relief, participants with above-average (26.5%) or excellent (44.9%) post-understanding were more likely to report that the VR session helped alleviate anxiety compared to those with an average understanding (8.7%) (*p* < 0.001). Participants who mentioned experiencing the VR in “first person” were more likely to report a stronger belief in the ability of VR to reduce anxiety about chemotherapy (40.5% vs. 23.7%, *p =* 0.033) (Table 7). The same result applied to those who mentioned myths (47.5% vs. 24.5%, *p* = 0.030).

The following quotes shared by participants offer a glimpse into how the participants felt and what they thought during the VR sessions, highlighting the effect of the simulation and the ability to reduce anxiety and uncertainty.

### 3.1. Reducing Anxiety

- “The experience let us have a clear understanding on what will occur during the first chemotherapy appointment and would take away some of the unknowns and answer questions that we may have going into it. Having more information and feeling educated about chemotherapy would greatly help take the anxiety out of a very anxious time.”
- “Seeing that space I would be in would be helpful to ease anxiety and understand where I’m going.”
- “Just knowing what to expect and where to go [greatly] lowers my anxiety in situations like these.”
- “Simply seeing and hearing the process beforehand. Nerves would already be a mess so having a place to turn for repeated info would be great.”
- “It’s way less stressful to approach the situation when you have already been virtually walked through all the steps beforehand.”

### 3.2. Previous Experience with Cancer

- “My mom is currently undergoing treatment. This would have been so helpful in the beginning of her treatment.”
- “I am familiar with chemo. I am positive people who are doing chemo for the first time will find it very useful.”
- “As a survivor I personally feel as though my anxiety surrounding my very different experience is always going to be there having already gone through the situation without the help of VR, but looking at this as an individual only recently diagnosed I believe that this could be put to wonderful use for future patients. It can ease anxiety by showing how manageable the illnesses often are and can equip individuals for things to expect while going through treatment.”
- “I had breast cancer and was able to take Exemestane for 5 years and [undergo] radiation. If I had this VR before it would have helped my anxiety.”

### 3.3. Understanding from a First-Person Perspective

- “The first person perspective was a powerful tool in managing my expectations and holding my attention.”
- “I truly felt the emotion of going to a new procedure, and it made me emotional to basically have someone holding my hand and walking me through the process so I wouldn’t be afraid or anxious.”
- “The first person perspective and being able to look around as if I’m the patient made everything feel more direct to me. I liked that I could go back and relisten if needed.”
- “This helps me understand what relatives in the past have experienced.”

### 3.4. Understanding

- “I didn’t understand all that was involved in the process before the video. Now I understand what can be expected for physical needs and potential emotions I would experience if I had to go through it.”
- “It was good to be able to go through the facility and receive information about the process on my own terms. Sometimes it is easier to process a lot of information on my own as opposed to in the physical presence of another person, especially in a medical setting.”
- “Compared to “internet knowledge”, [it] gave me a better understanding of chemotherapy experience.”

### 3.5. Enabling and Equipping

- “Seeing the facilities in advance, knowing the route to take, seeing the kindness and the patience of the staff, and understanding such an invasive/sensitive process before you’re living it for the first time–all of those factors could make the lived experience easier.”
- “It automatically took me to the comfort level I’d have going into the second appointment. I already feel as though I have one appointment under my belt, and I don’t feel as scared.”
- “Anxiety-provoking situations are as such because they are experiences that are unfamiliar, and there’s a general fear of the unknown or change; however, VR allowed me to be more equipped to face, familiar with, and informed about the chemotherapy process, reducing the anxiety.”

### 3.6. Complaints

- “The audio was not great, and I think it would be better with clearer instructions.”
- “The presence of other people in the room was distracting and confusing.”
- “I think the VR tour lost some [of] its immersion/realism due to moments where the video blurred.”
- “The only note I really had was that their (sic) were some noises in the VR experience that made me a bit uneasy… I do absolutely think that being provided with information about the process in this way was helpful, it was just the external factors that made it a bit anxiety inducing.”

### 3.7. Realistic Experience

- “It would have been helpful back when I received treatment as it sets realistic expectations.”
- “Everything… this was very realistic.”

The study’s key findings encompass a comprehensive understanding of the demographic landscape and the impact of VR experiences on various post-intervention variables related to the understanding of chemotherapy, anxiety, and expectations of VR. The VR intervention positively influenced participants’ understanding of chemotherapy, with a significant increase in post-intervention scores. Conversely, the realism and usefulness of the VR experience were influenced by gender, ethnicity, and age, with notable variations observed in our data. Emotional responses and feelings of control during VR sessions correlated with post-intervention variables, indicating their potential impact on anxiety reduction and expectations. Qualitative analyses further highlighted associations between participants’ experiences during VR sessions and post-understanding, anxiety, and expectations, emphasizing the multifaceted nature of VR’s impact in medical settings.

## 4. Discussion

Our study explored the impact of an immersive virtual tour on increasing understanding and reducing anxiety about the chemotherapy process. Consistent with previous research, our study demonstrated a significant increase in participants’ understanding of chemotherapy processes following exposure to the VR intervention [11,27,28]. Moreover, gender, ethnicity, and age emerged as influential factors shaping perceptions of VR realism and usefulness, echoing the findings of previous studies [9,10,28,48]. These findings resonate with the existing literature, emphasizing the importance of understanding demographic factors in shaping perceptions and experiences of VR interventions [9,11,27,28,29]. The findings also underscore the necessity of considering diverse patient backgrounds in designing and implementing VR interventions to ensure their efficacy, as well as provide a broader impact to a diverse subset of patients. 

Qualitative analyses provided additional insights into the associations between participants’ experiences during VR sessions and post-intervention variables, emphasizing the multifaceted nature of VR’s impact in medical contexts. These qualitative findings align with the existing literature, highlighting the importance of subjective experiences in shaping perceptions and outcomes [12,29].

Our results highlight the potential of VR in alleviating anxiety and enhancing the treatment experience for cancer patients and their caregivers, corroborating findings from prior research [9,10,28,48]. The immersive nature of VR, coupled with its ability to provide a sense of control, offers promising avenues for addressing psychological distress during medical procedures. However, it is essential to acknowledge the need for further research to establish the long-term effects of VR interventions and optimize their integration into clinical practice [4,27]. Overall, our study contributes to the growing body of evidence supporting the efficacy of VR interventions in medical education, anxiety management, and treatment enhancements in chemotherapy.

By leveraging immersive technologies, healthcare practitioners can better engage patients and caregivers, improve their understanding, and alleviate psychological distress, ultimately enhancing patient outcomes and quality of care. VR technology creates new possibilities for patient experience and satisfaction. Using VR to provide educational resources to patients not only increases their understanding of treatment information but can also change the future of healthcare. The use of VR technology in rural or understaffed healthcare settings is something to be considered. Using VR can be an inexpensive option and provides various opportunities for education and patient care [3].

Our study’s findings align with previous studies, which found that participants who viewed the VR intervention versus a pre-recorded video had a better understanding and knowledge of radiation therapy [9]. It is believed that using visuals, such as viewing the procedure, and immersion in VR allows participants to remain engaged with the information and improve retention [9]. We also found that participants who experienced VR from a “first person” perspective or held pre-existing myths about chemotherapy reported higher levels of anxiety (*p* = 0.033 and *p* = 0.030, respectively). This contrasts with the generally positive outcomes reported in prior studies. It suggests that while VR can reduce anxiety for many, it may also exacerbate it for some individuals, particularly those with preconceived fears or misconceptions. This nuance is less commonly addressed in the existing literature, which typically focuses on the broad benefits of VR without delving into these potential adverse effects.

It is important to recognize the various limitations of this study. Primarily, the participant pool was predominantly composed of college students, faculty, and staff recruited via university email lists. This convenience sample restricts the generalizability of the findings, particularly to populations outside a rural Appalachian setting. Future studies should aim to include a more diverse range of participants from various locations and backgrounds to enhance the applicability of the results. Additionally, the study involved only 26 participants who experienced the VR simulation in person using headsets. This small sample size limits the depth of understanding regarding the potential effects of VR interventions. Future research should aim to include a larger number of in-person participants to gain a more comprehensive and nuanced understanding of VR’s impact on patient education and anxiety reduction. Furthermore, some survey questions in pre- and post-interventions may have been ambiguously phrased, which could have affected the accuracy of participant responses. Future studies should focus on refining and clarifying these questions to ensure they accurately capture the intended information and provide reliable data. Due to funding limitations, the equipment used to capture the experience was limited, and the headsets used for viewing the experience were not of the highest quality. Additionally, access to the hospital was limited, imposing certain restrictions on the study. Despite these limitations, the study provides valuable insights into the potential benefits of VR for enhancing patient education and reducing anxiety related to chemotherapy. It highlights the importance of conducting research with more diverse populations and improving methodological rigor to better understand and utilize VR technology in healthcare settings.

## 5. Conclusions

Virtual reality (VR) and 360-degree technology are transforming the medical field, introducing new possibilities for patient education and treatment. Recent studies have shown the practicality and benefits of VR technology in cancer therapy, such as reducing treatment symptoms, offering educational support, and enhancing the quality of life for chemotherapy patients. A key advantage of using VR to support cancer treatment is its immersive nature, which helps divert the patient’s attention from the discomfort and stress often associated with cancer treatment. Furthermore, VR applications in cancer care can assist healthcare professionals and caregivers in their roles, bridging gaps in explanation and comprehension between healthcare workers and patients. Consistent explanations tailored to the patient throughout all treatments will successfully ensure patients are equipped with the suitable information to answer their questions. With a shared understanding of treatment and the process between the patients and healthcare professionals, more time can be spent focusing on the support of the patient. Additionally, VR-based tools can bolster caregiver education and support by equipping them with resources to better understand and cater to the needs of the individual going through cancer treatment. The results of this study showcase that VR and 360-degree technology have the potential to serve as tools for enhancing understanding and reducing anxiety related to chemotherapy treatments. With the utilization of “pre-reality”, patients will be more equipped with an insight into what their first day of chemotherapy will entail. With this increased understanding, the anxiety that accompanies the unknown will be reduced, relieving the patients from a subset of their distress and granting them more time and energy to use for improving their health.

## Figures and Tables

**Table 1:** Characteristics of Survey Participants by Video View.

	360-Degree Video	VR Headset	
	(*n* = 241)	(*n* = 26)	
Demographics	*n* (Percent) ϯ	*n* (Percent) ϯ	*p*
Age Category (In Years)			0.412 *
18–29	110 (45.6)	13 (50.0)	
30–39	70 (29.0)	5 (19.2)	
40–49	29 (12.0)	7 (26.9)	
50–59	16 (6.6)	1 (3.8)	
60+	16 (6.6)	0 (0.0)	
Gender			
Male	55 (22.8)	13 (50.0)	0.004 **
Female	176 (73.0)	13 (50.0)	
Transgender	3 (1.2)	0 (0.0)	
Other	6 (2.5)	0 (0.0)	
Prefer Not to Answer	1 (0.4)	0 (0.0)	
Race and Ethnicity ✢			
Native American or Alaskan Native	1 (0.4)	1 (3.8)	
Asian	17 (7.1)	1 (3.8)	
Black or African American	34 (14.1)	0 (0.0)	
Hispanic of Any Race	11 (4.6)	2 (7.7)	
White	178 (73.9)	23 (88.5)	
Other	5 (2.1)	0 (0.0)	
Prefer Not to Answer	5 (2.1)	0 (0.0)	
Level of Education			
Primary	0 (0.0)	0 (0.0)	
High School	42 (17.4)	5 (19.2)	
Pre-University	43(17.8)	2 (7.7)	
Graduate	116 (48.1)	14 (53.8)	
Post-Graduate	40 (16.6)	5 (19.2)	
Employment Status			0.782
Employed	189 (78.4)	21 (80.8)	
Unemployed	52 (21.6)	5 (19.2)	
Cancer Survivor?			0.143 ***
Yes	23 (9.5)	0 (0.0)	
No	218 (90.3)	26 (100.0)	
Loved One or Close Friend with Cancer			0.279 ***
Yes	199(82.9)	19 (73.1)	
No	41 (17.1)	7 (26.9)	
Viewing Technology			
Cell Phone	113 (46.9)	4 (15.4)	
Tablet	10 (4.1)	0 (0.0)	
Laptop Computer	90 (37.3)	0 (0.0)	
Desktop Computer	22 (9.1)	0 (0.0)	
Virtual Reality Headset	6 (2.5)	22 (84.6)	

Ϯ Note: Percentages are based on non-missing values. ✢ Note: Respondents could select all that apply, creating values that equal >100 percent. * Note: Pearson correlation was used for *p*-value calculations due to the continuous nature of the age variable. ** Note: Chi-Square was calculated between male and female respondents due to small expected cell counts. *** Note: Fisher’s exact test was used for *p*-value calculations due to small sample sizes.

**Table 2:** Results Comparing Demographic Variables and Post-intervention VR Encounters.

Variable	Category/ Measure	Group A *n* (Percent)	Group B *n* (Percent)	Statistic	*p*
Categorical Variables					
Perception of Chemotherapy	Stays the Same	Males: 12 (18.2)	Females: 61 (33.2)	χ^2^ = 5.266	0.022
	More Positive	Males: 54 (81.8)	Females: 123 (66.8)		
Change in Emotional/Physical Understanding of Chemotherapy	Yes	Black: 17 (50.0)	Non-Black: 161 (69.7)	χ^2^ = 5.214	0.022
	No	Black: 17 (50.0)	Non-Black: 70 (30.3)		
Realism of VR	Average	White: 36 (18.4)	Non-White: 20 (31.3)	χ^2^ = 4.738	0.030
	Above Average	White: 196 (81.6)	Non-White: 64 (68.8)		
VR Useful Tool in Managing Anxiety	Somewhat	White: 23 (11.6)	Non-White: 13 (19.7)	χ^2^ = 6.052	0.049
	Moderately So	White: 71 (35.7)	Non-White: 29 (43.9)		
	Very Much So	White: 105 (52.8)	Non-White: 24 (36.4)		
Continuous Variables					
Age x Realism of VR	Mean ± SD	Age: 33.4 ± 13.2	Realism: 4.1 ± 0.8	*r_s_*(265) = 0.132	0.032

**Table 3:** Results Comparing VR Experiences and Post-intervention Variables.

Variable	Category/ Measure	Group A *n* (Percent)	Group B *n* (Percent)	Group C *n* (Percent)	Statistic	*p*
Understanding						
Perception of Chemotherapy	Stays the Same	Average 23 (41.8)	Above Average 40 (27.4)	Excellent 11 (21.2)	χ^2^ = 6.087	0.048
	More Positive	Average 32 (58.2)	Above Average 106 (72.6)	Excellent 41 (78.8)		
Reduce Anxiety						
Perception of Chemotherapy	Stays the Same	Somewhat 54 (31.8)	To a Great Extent 3 (4.9)		χ^2^ = 17.408	<0.001
	More Positive	Somewhat 116 (68.2)	To a Great Extent 58 (95.1)			
Manage Expectations about Chemotherapy						
Perception of Chemotherapy	Stays the Same	Somewhat 52 (36.6)	To a Great Extent 18 (16.7)		χ^2^ = 12.114	<0.001
	More Positive	Somewhat 90 (63.4)	To a Great Extent 90 (83.3)			
Understanding						
Change in Emotional/Physical Understanding of Chemotherapy	Yes	Average 28 (50.0)	Above Average 108 (72.5)	Excellent 36 (69.2)	χ^2^ = 9.452	0.009
	No	Average 28 (50.0)	Above Average 41 (27.5)	Excellent 16 (30.8)		
Reduce Anxiety						
Change in Emotional/Physical Understanding of Chemotherapy	Yes	Somewhat 110 (64.0)	To a Great Extent 52 (85.2)		χ^2^ = 9.636	0.002
	No	Somewhat 62 (36.0)	To a Great Extent 9 (14.8)			
Manage Expectations about Chemotherapy						
Change in Emotional/Physical Understanding of Chemotherapy	Yes	Somewhat 88 (62.0)	To a Great Extent 87 (78.4)		χ^2^ = 7.864	0.005
	No	Somewhat 54 (38.0)	To a Great Extent 24 (21.6)			

**Table 4:** Results Comparing VR Characteristics and Post-intervention Variables.

Variable	Category/ Measure	Group A *n* (Percent)	Group B *n* (Percent)	Group C *n* (Percent)	Statistic	*p*
Understanding						
VR Useful Tool in Managing Anxiety	Somewhat	Average 15 (27.3)	Above Average 17 (11.3)	Excellent 2 (3.8)	*r_s_*(265) = 0.263	<0.001
	Moderately So	Average 24 (43.6)	Above Average 58 (38.4)	Excellent 16 (30.8)		
	Very Much So	Average 16 (29.1)	Above Average 76 (50.3)	Excellent 34 (65.4)		
Reduce Anxiety						
VR Useful Tool in Managing Anxiety	Somewhat	Somewhat 17 (9.9)	To a Great Extent 2 (3.2)		*r_s_*(234) = 0.462	<0.001
	Moderately So	Somewhat 89 (51.7)	To a Great Extent 2 (3.2)			
	Very Much So	Somewhat 66 (38.4)	To a Great Extent 58 (93.5)			
Manage Expectations about Chemotherapy						
VR Useful Tool in Managing Anxiety	Somewhat	Somewhat 30 (21.1)	To a Great Extent 2 (1.8)		*r_s_*(2538) = 0.238	<0.001
	Moderately So	Somewhat 73 (51.4)	To a Great Extent 23 (20.5)			
	Very Much So	Somewhat 39 (27.5)	To a Great Extent 87 (77.7)			
Understanding						
Control of Situation after VR Tour	A Little	Average 13 (23.6)	Above Average 11 (7.3)	Excellent 2 (3.8)	*r_s_*(264) = 0.323	<0.001
	Moderately	Average 24 (43.6)	Above Average 61 (40.7)	Excellent 16 (30.8)		
	Quite a Bit	Average 18 (32.7)	Above Average 78 (52.0)	Excellent 34 (65.4)		
Reduce Anxiety						
Control of Situation after VR Tour	A Little	Somewhat 15 (8.7)	To a Great Extent 1 (1.6)		*r_s_*(233) = 0.436	<0.001
	Moderately	Somewhat 83 (48.3)	To a Great Extent 6 (9.8)			
	Quite a Bit	Somewhat 74 (43.0)	To a Great Extent 54 (88.5)			
Manage Expectations about Chemotherapy						
Control of Situation after VR Tour	A Little	Somewhat 19 (13.4)	To a Great Extent 2 (1.8)		*r_s_*(257) = 0.318	<0.001
	Moderately	Somewhat 75 (52.8)	To a Great Extent 29 (25.9)			
	Quite a Bit	Somewhat 48 (33.8)	To a Great Extent 81 (72.3)			
Understanding						
Clear Image of Chemotherapy After VR Tour	Unsure	Average 7 (13.2)	Above Average 3 (2.0)	Excellent 1 (1.9)	*r_s_*(265) = 0.381	<0.001
	Helpful	Average 35 (66.0)	Above Average 90 (60.8)	Excellent 16 (30.8)		
Reduce Anxiety						
Clear Image of Chemotherapy After VR Tour	Unsure	Somewhat 6 (3.6)	To a Great Extent 0 (0.0)		*r_s_*(233) = 0.362	<0.001
	Helpful	Somewhat 162 (96.4)	To a Great Extent 61 (100.0)			
Manage Expectations about Chemotherapy						
Clear Image of Chemotherapy After VR Tour	Unsure	Somewhat 9 (6.4)	To a Great Extent 0 (0.0)		*r_s_*(258) = 0.206	<0.001
	Helpful	Somewhat 131 (93.6)	To a Great Extent 112 (100.0)			
Understanding						
Likelihood of Recommending VR for Other Procedures	Average	Average 22 (41.5)	Above Average 29 (19.3)	Excellent 2 (3.8)	*r_s_*(262) = 0.261	<0.001
	Above Average	Average 31 (58.5)	Above Average 121 (80.7)	Excellent 50 (96.2)		
Reduce Anxiety						
Likelihood of Recommending VR for Other Procedures	Average	Somewhat 43 (25.3)	To a Great Extent 1 (1.6)		*r_s_*(232) = 0.314	<0.001
	Above Average	Somewhat 127 (74.7)	To a Great Extent 60 (98.4)			
Manage Expectations about Chemotherapy						
Likelihood of Recommending VR for Other Procedures	Average	Somewhat 37 (26.6)	To a Great Extent 14 (12.5)		*r_s_*(255) = 0.331	<0.001
	Above Average	Somewhat 102 (73.4)	To a Great Extent 98 (87.5)			
Understanding						
Realism of VR Tour	Average	Average 22 (41.5)	Above Average 29 (19.3)	Excellent 2 (3.8)	*r_s_*(258) = 0.314	<0.001
	Above Average	Average 31 (58.5)	Above Average 121 (80.7)	Excellent 50 (96.2)		
Reduce Anxiety						
Realism of VR Tour	Average	Somewhat 41 (25.3)	To a Great Extent 1 (1.6)		*r_s_*(234) = 0.375	<0.001
	Above Average	Somewhat 127 (74.7)	To a Great Extent 60 (98.4)			
Manage Expectations about Chemotherapy						
Realism of VR Tour	Average	Somewhat 37 (26.6)	To a Great Extent 14 (12.5)		*r_s_*(254) = 0.350	<0.001
	Above Average	Somewhat 102 (73.4)	To a Great Extent 98 (87.5)			

**Table 5:** Qualitative Themes, Definitions, and Exemplars.

Theme	Definition	Exemplar Quote
VR Experience	Captures the overall user experience, including first-person perspective, staff support, realism, and side effects like dizziness.	“The first person perspective was a powerful tool in managing my expectations and holding my attention.”
Knowledge	Information conveyed through the VR experience, such as the chemotherapy process, treatment steps, and addressing misconceptions.	“I didn’t understand all that was involved in the process before the video. Now I understand what can be expected for physical needs and potential emotions.”
Environment	The physical setting portrayed in the VR experience, including the surroundings, space, and available resources.	“Seeing that space I would be in would be helpful to ease anxiety and understand where I’m going.”
Emotional Impact	The emotional responses elicited by the VR experience, particularly in terms of relieving or inducing anxiety.	“It’s way less stressful to approach the situation when you have already been virtually walked through all the steps beforehand.”
Improvements	Areas for enhancement in the VR experience, including technical aspects and content-related suggestions.	“I think the VR tour lost some [of] its immersion/realism due to moments where the video blurred.”
Overall Reactions	Summarizes participants’ positive and negative reactions to the VR experience.	“Everything... this was very realistic.”

**Table 6:** Qualitative Codes by Video View.

	360-Degree Video	VR Headset	
	(*n* = 241)	(*n* = 26)	
Qualitative Codes	*n* (Percent) ϯ	*n* (Percent) ϯ	*p*
Experience	133 (55.2)	16 (61.5)	0.536
First Person	36 (14.9)	6 (23.1)	0.265 *
Friendliness/Support of Staff	20 (8.3)	5 (19.2)	0.080 *
Realistic	53 (22.0)	3 (11.5)	0.214
VR Side Effects/Dizziness	4 (1.7)	6 (23.1)	<0.001
Speaking About the Nurse	80 (33.2)	12 (46.2)	0.187
Knowledge	189 (78.4)	23 (88.5)	0.229
Process	140 (57.7)	15 (58.1)	0.969
Explaining Chemo/Treatment	65 (27.0)	8 (30.8)	0.68
Symptoms/Side Effects	57 (23.7)	11 (42.3)	0.038
Q&A	66 (27.4)	7 (26.9)	0.96
Eliminated Myths/Misconceptions	19 (7.9)	3 (11.5)	0.459 *
Environment	91 (34.6)	9 (37.8)	0.753
Pre-nurse (Prior to Seeing Nurse)	21 (8.7)	1 (3.8)	0.706 *
Surrounding/Space	71 (29.5)	6 (23.1)	0.65
Resources Provided (e.g., Snacks)	10 (4.1)	2 (7.7)	0.330 *
Emotional	209 (86.7)	25 (96.2)	0.220 *
Relieved Anxiety	207 (85.9)	25 (96.2)	0.220 *
Increased Anxiety	7 (2.9)	4 (15.4)	0.015 *
Improvements	76 (31.5)	19 (73.1)	<0.001
VR Video	30 (12.4)	14 (53.8)	<0.001
Content	56 (23.2)	10 (38.5)	0.087
Tailored VR Experience	10 (4.1)	0 (0.0)	
Simulation of Treatment	27 (11.2)	7 (26.9)	0.032 *
All			
Positive	221 (91.7)	26 (100)	0.235 *
Negative	79 (32.8)	20 (76.9)	<0.001

ϯ Note: Percentages are based on non-missing values. * Note: Fisher’s exact test was used for *p*-value calculations due to small expected cell counts.

**Table 7:** Results Comparing Qualitative Codes and Post-intervention VR Encounters.

Variable	Category/ Measure	Group A *n* (Percent)	Group B *n* (Percent)	Statistic	*p*
Categorical Variables					
Process					
Understanding	Average	Yes 24 (15.7)	No 32 (30.2)	χ^2^ = 8.218	0.016
	Above Average	Yes 98 (64.1)	No 53 (50.0)		
	Excellent	Yes 31 (20.3)	No 21 (19.9)		
Perception of Chemotherapy	Stays the Same	Yes 39 (25.7)	No 38 (35.2)	χ^2^ = 2.749	0.097
	More Positive	Yes 113 (74.3)	No 70 (64.8)		
How Much Did VR Reduce Anxiety	Somewhat	Yes 99 (71.7)	No 74 (76.3)	χ^2^ = 0.607	0.436
	To a Great Extent	Yes 39 (28.3)	No 23 (23.7)		
Emotions					
Understanding	Average	Yes 44 (19.2)	No 12 (40.0)	χ^2^ = 6.801	0.033
	Above Average	Yes 138 (60.3)	No 13 (43.3)		
	Excellent	Yes 47 (20.5)	No 5 (16.7)		
Perception of Chemotherapy	Stays the Same	Yes 63 (27.5)	No 14 (45.2)	χ^2^ = 4.081	0.043
	More Positive	Yes 166 (72.5)	No 17 (54.8)		
How Much Did VR Reduce Anxiety	Somewhat	Yes 151 (72.2)	No 22 (84.6)	χ^2^ = 1.821	0.177
	To a Great Extent	Yes 58 (27.8)	No 4 (15.4)		
Reduce Anxiety					
Understanding	Average	Somewhat 42 (91.3)	To a Great Extent 4 (8.7)	χ^2^ = 15.859	<0.001
	Above Average	Somewhat 100 (73.5)	To a Great Extent 36 (26.5)		
	Excellent	Somewhat 27 (55.1)	To a Great Extent 22 (44.9)		
First Person					
Understanding	Average	Yes 7 (16.7)	No 49 (22.6)	χ^2^ = 3.824	0.148
	Above Average	Yes 22 (52.4)	No 129 (59.4)		
	Excellent	Yes 13 (31.0)	No 39 (18)		
Perception of Chemotherapy	Stays the Same	Yes 10 (23.8)	No 67 (30.7)	χ^2^ = 0.810	0.368
	More Positive	Yes 32 (76.2)	No 151 (69.3)		
How Much Did VR Reduce Anxiety	Somewhat	Yes 22 (59.5)	No 151 (76.3)	χ^2^ = 4.532	0.033
	To a Great Extent	Yes 15 (40.5)	No 47 (23.7)		
Myths					
Understanding	Average	Yes 3 (14.3)	No 53 (22.3)		0.394 *
	Above Average + Excellent	Yes 18 (85.7)	No 185 (77.7)		
Perception of Chemotherapy	Stays the Same	Yes 4 (19.0)	No 73 (30.5)	χ^2^ = 1.224	0.269
	More Positive	Yes 17 (81.0)	No 166 (69.5)		
How Much Did VR Reduce Anxiety	Somewhat	Yes 10 (52.6)	No 163 (75.5)	χ^2^ = 4.687	0.030
	To a Great Extent	Yes 9 (47.4)	No 53 (24.5)		

* Note: Fisher’s exact test was used for *p*-value calculations due to small sample sizes.

## Data Availability

The datasets presented in this article are not readily available because the data are part of an ongoing study. Requests to access the datasets should be directed to Melissa Thomas.
